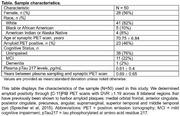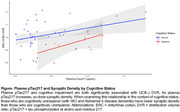# Associations between plasma pTau217, cognitive impairment, and synaptic density in AD vulnerable brain regions

**DOI:** 10.1002/alz70856_103318

**Published:** 2025-12-26

**Authors:** Kao Lee Yang, Alexandra H DiFilippo, Yazan Hammad, Yue Ma, Rachael E. Wilson, Mary‐Elizabeth Pasquesi, Erin M. Jonaitis, Todd E Barnhart, Jonathan W Engle, Tobey J. Betthauser, Nicholas Ashton, Sterling C Johnson, Bradley T Christian, Henrik Zetterberg, Barbara B. Bendlin

**Affiliations:** ^1^ Wisconsin Alzheimer's Disease Research Center, University of Wisconsin School of Medicine and Public Health, Madison, WI, USA; ^2^ Department of Medical Physics, University of Wisconsin, Madison, WI, USA; ^3^ Wisconsin Alzheimer's Institute, University of Wisconsin School of Medicine and Public Health, Madison, WI, USA; ^4^ Wisconsin Alzheimer's Disease Research Center, University of Wisconsin‐Madison School of Medicine and Public Health, Madison, WI, USA; ^5^ Wisconsin Alzheimer's Institute, University of Wisconsin‐Madison School of Medicine and Public Health, Madison, WI, USA; ^6^ Department of Psychiatry and Neurochemistry, Institute of Neuroscience and Physiology, The Sahlgrenska Academy, University of Gothenburg, Mölndal, Sweden; ^7^ Geriatric Research Education and Clinical Center (GRECC), William S. Middleton Memorial Veterans Hospital, Madison, WI, USA; ^8^ Wisconsin Alzheimer's Institute, University of Wisconsin School of Medicine and Public Health, Madison, WI, USA; ^9^ Hong Kong Center for Neurodegenerative Diseases, Hong Kong, Science Park, China; ^10^ Department of Neurodegenerative Disease, UCL Institute of Neurology, Queen Square, London, United Kingdom; ^11^ UK Dementia Research Institute, University College London, London, United Kingdom; ^12^ Department of Psychiatry and Neurochemistry, Institute of Neuroscience and Physiology, the Sahlgrenska Academy at the University of Gothenburg, Mölndal, Gothenburg, Sweden; ^13^ Wisconsin Alzheimer's Disease Research Center, School of Medicine and Public Health, University of Wisconsin‐Madison, Madison, WI, USA

## Abstract

**Background:**

Phosphorylated tau and amyloid are known to cluster in synaptosomes in Alzheimer's disease (AD). Their presence in synapses may contribute to synaptic loss, although this relationship is not well understood. Plasma phosphorylated tau at position 217 (pTau217) represents tau secreted from neurons, is an accurate biomarker of AD pathology, and could potentially shed light on the processes preceding synaptic loss and cognitive impairment. Here we examine this relationship in medial‐temporal brain regions susceptible to AD pathology accumulation.

**Methods:**

This analysis included 50 participants enrolled in the Wisconsin Alzheimer's Disease Research Center and the Wisconsin Registry for Alzheimer's Prevention with plasma data and [C‐11]UCB‐J PET scans (Table). Plasma pTau217 was determined using the ALZpath pTau217 Simoa assay on Quanterix HD‐X platform. We used Logan Graphical Analysis with a whole cerebellar reference region to quantify synaptic density from [C‐11]UCB‐J scans, and identified ROIs (entorhinal cortex, hippocampus, fusiform gyrus, parahippocamphal gyrus, amygdala, and temporal pole) using *FreeSurfer* T1w‐MRI parcellation. Amyloid positivity was determined using [C‐11]PiB PET scans with a global DVR index ^3^1.19. We utilized multiple regression analysis to examine the extent to which plasma pTau217 and whether having cognitive impairment associated with synaptic density in ROIs controlling for age and amyloid status. All models were fitted in R and considered significant at unadjusted *p* <.05. Effect sizes were assessed using Cohen's f^2^.

**Results:**

Higher levels of plasma pTau217 (b=.13, *p* = 0.02) associated with higher synaptic density in the entorhinal cortex and cognitive impairment (b=‐.09, *p* = 0.02) associated with lower synaptic density in this same region with small‐to‐moderate effect sizes (f^2^=.13 and f^2^=.12, respectively; see Figure). Cognitive impairment was associated with lower UCB‐J DVR in the hippocampus (b=‐.07, *p* <.05) with a moderate‐to‐large effect size of f^2^=.31.

**Conclusions:**

The unexpected directional relationship between pTau217 and synaptic density in the medial temporal lobe could be due to an early compensatory response to pathology accumulation since most individuals in this sample were cognitively unimpaired. Longitudinal studies are forthcoming to determine the trajectory of plasma pTau217 and synaptic density.